# Small RNAs, Big Diseases

**DOI:** 10.3390/ijms21165699

**Published:** 2020-08-09

**Authors:** Iwona Rzeszutek, Aditi Singh

**Affiliations:** 1 Institute of Biology and Biotechnology, Department of Biotechnology, University of Rzeszow, Pigonia 1, 35-310 Rzeszow, Poland; 2 Max Planck Institute for Developmental Biology, Max-Planck-Ring 5, 72076 Tübingen, Germany

**Keywords:** noncoding RNA, miRNAs, chromosome fragile sites (CFSs), chromosome rearrangements, diseases

## Abstract

The past two decades have seen extensive research done to pinpoint the role of microRNAs (miRNAs) that have led to discovering thousands of miRNAs in humans. It is not, therefore, surprising to see many of them implicated in a number of common as well as rare human diseases. In this review article, we summarize the progress in our understanding of miRNA-related research in conjunction with different types of cancers and neurodegenerative diseases, as well as their potential in generating more reliable diagnostic and therapeutic approaches.

## 1. Introduction

The discovery of noncoding RNAs (ncRNAs) [1] has not only challenged the central dogma but also has brought RNA biology to the forefront in the understanding of almost all cellular processes within a cell [2,3,4,5,6]. ncRNAs are divided into two groups based on their transcript length, namely, small (sncRNA; < 200 nucleotides) and long (lncRNA; > 200 nucleotides) noncoding RNA. These ncRNAs form the RNA-infrastructure [7] that are involved not only in the processing of other RNAs such as mRNAs, tRNAs, and rRNAs but also in gene regulation by targeting mRNAs and chromatin [8,9,10,11].

The first tiny regulatory RNAs discovered to play an important role in the gene expression were *lin-4* RNA and *let-7* RNA, which control the cell fate transition through the larval development in worms [1,12,13,14]. These two short molecules are now the fundamental members of a class of noncoding RNA termed microRNAs (miRNAs), which are endogenous single-stranded, 18–25 nucleotides (nt) long RNA molecules [15]. Since then, miRNAs have been shown to have regulatory functions in most eukaryotes affecting cell growth, development, and differentiation [16,17,18].

## 2. MiRNAs Biogenesis and Function

Canonical biogenesis of miRNAs starts in the nucleus with the transcription of DNA sequences into primary miRNAs (pri-miRNAs) by RNA Polymerase II/III [3]. Next, these pri-miRNAs (characterized by stem-loop structures with a 3′ poly-A-tail and 5′ methylated cap) are processed by the Microprocessor complex. The Microprocessor complex composed of Drosha (an RNase III endonuclease) and DiGeorge Syndrome Critical Region 8 (DGCR-8) cleaves pri-miRNA to produce 60–70 nt precursor miRNAs (pre-miRNAs) [19]. 

Pre-miRNA may also be produced by the noncanonical process, where small RNAs are produced from short intronic hairpins termed “mirtrons,” and unlike the canonical mechanism, it does not require Drosha for pre-miRNA production [20,21].

Once pri-miRNAs are generated, they are exported to the cytoplasm in an Exportin5-/RanGTP-dependent manner where they are processed by another RNase III enzyme—Dicer, to produce mature miRNA [22]. The functional strand of mature miRNA is subsequently loaded into the Argonaut (AGO) family proteins to form a miRNA-induced silencing complex (miRISC) [23]. Mature miRNA usually binds to the “seed” region (5–8 nt long) in the 3′ UTR of the target mRNA [24]. However, other sequences such as 5′ UTR, coding region, or gene promoters have also been reported as miRNA-binding sites [25,26,27]. The sequence complementarity via miRNA works leads to degradation, destabilization, or translational repression [28,29].

The majority of miRNA sequences are located in introns of noncoding or coding transcripts, although some miRNAs may overlap with exons [30]. Interestingly, up to 2000 miRNAs have been identified in *Homo sapiens* alone, which are involved in direct regulation of more than 60% of protein-coding genes [31], suggesting that a single miRNA can regulate expression more than a hundred mRNAs [32]. Therefore, any aberrant regulation and misfunction of miRNAs eventually cause various disease conditions [33], which we would like to discuss in more detail in the following sections.

## 3. miRNA Located in Genomic Regions Prone to Rearrangements

Numerous studies indicate that miRNAs distribution is not randomly organized in the human genome. It has been shown that some of the chromosomes have higher numbers of miRNAs than the others. Some of the earliest chromosomes discovered first with higher numbers of miRNA are chromosomes (chr.) 1, 2, 19, and X. [34,35]. However, since the number of known miRNAs is expanding continuously, recent data indicates that chr. 14, 16, 17, 22, and X are also abundant with miRNAs with chr. 19 having the highest number in comparison to others [36]. Interestingly, chromosomes that are abundant with miRNAs also have the highest gene densities [37,38], high minisatellites number [39], as well as high expression level [40]. Furthermore, most of the chromosomes abundant with miRNAs are also prone to a higher rate of mutations and are linked to a variety of diseases [41,42,43,44]. Chromosomal fragile sites (CFS) and cancer-associated genomic regions (CAGR) are widely studied examples of such regions.

### 3.1. miRNA Located at Chromosomal Fragile Sites (CFSs)

Chromosomal fragile sites (CFSs) are specific chromosomal regions (cover 26.38% of human chromosomes [45]) prone to breakage and rearrangements when cells are exposed to DNA replication inhibitors [46]. CFSs are highly transcribed sequences, conserved across the genomes of different eukaryotes such as yeast *S. cerevisiae,* mouse, rat, and many mammals, including humans [47,48,49]. These specific sites are defined as “rare” and “common” based on their frequency [50,51]. Most “rare” fragile sites can be induced by bromodeoxyuridine (BrdU) or by the removal of folic acid, whereas most “common” fragile sites are induced by aphidicolin or 5-azacytidine [52,53]. CFSs are often characterized by the presence of repetitive sequences. “Rare” CFSs are mostly associated with CCG/CGG trinucleotide repeat sequences adjacent to a CpG island [54], whereas “common” CFSs are located at AT-rich minisatellite repeats [55]. Nevertheless, CFSs may also embody other repetitive elements such as LINE1 and LINE2, Alu, MIR, and MER, as well as endogenous retroviral sequences [56]. Interestingly, some mammalian miRNAs are derived from genomic repeats. For instance, some of them show perfect complementarity to the MIR/LINE-2 class of repeat elements [57].

The abundance of miRNA on fragile sites differs among chromosomes. Lagana et al. have shown that chromosomes 16, 19, and X are abounding in miRNAs at the fragile sites. Unlike these chromosomes, chr. 14 shows the opposite results (e.g., less abundant miRNAs in fragile regions) [58].

The Human Database currently documents 125 fragile sites (containing 4921 protein-coding genes) lying in both somatic chromosomes and the sex chromosome X. Analysis performed by Kumar et al. [59] indicate that 34.51% of human protein-coding genes lie within the CFSs showing the importance of stability of fragile sites in proper gene expression.

### 3.2. miRNA Located at the Cancer-Associated Genomic Regions (CAGRs)

Another region prone to rearrangements where miRNA is frequently present is cancer-associated genomic regions (CAGR) [34,60,61]. CAGRs are characterized by (i) minimal regions of loss of heterozygosity (LOH), suggestive of the presence of tumour suppressor genes; (ii) minimal regions of amplification, suggestive of the presence of oncogenes; and (iii) common breakpoint regions in or near possible oncogenes or tumour suppressor genes. The frequency of miRNAs localized in these regions is 52.5% [34]. For instance, miR-21, miR155, and miR17-92 cluster are amplified CAGRs [62] expressed at a much higher level in tumour cells [63].

### 3.3. Relationship between Higher-Order Chromosomal Structure and miRNAs

It has been previously shown that the three-dimensional (3D) organization of the genome contributes to the genome rearrangements and translocations genome-wide [64]. However, the relationship between miRNA and genomic structure has not yet been fully explored. Recent data indicate that the 3D architecture of chromatin influences the transcription of microRNA genes (MIRs) [65]. Chen et al. [65] have shown that miRNAs possess features similar to protein-coding genes; both undergo coordinated expression through their chromosomal loci interactions. It has been shown that a substantial number of miRNAs are controlled by cis genetic regulatory elements, such as CpG islands (2%), promoters (9%), enhancers (35%), and transcription factor (TF) binding regions (15%), which may affect miRNAs expression level [66]. Additionally, the analysis performed on a large number of breast cancer samples has shown that, to some extent, miRNAs and their neighbouring genes may have a positive correlative expression [67].

Beside small noncoding RNAs, long intergenic noncoding RNAs (lincRNAs), class of long noncoding RNAs, have also been shown to influence transcriptional regulation through their long-range chromatin interactions [68]. Like microRNA, most of the lincRNAs interacts with protein-coding genes (two or more) [68]. Furthermore, studies performed by Cai et al. [68] have shown that numerous lincRNA promoters were linked with a higher state of enhancer-like chromatin with a higher level of H3K4me1 compared to H3K4me3, corroborating with previous studies [69,70]. Interestingly, most recent data have shown that AGO1, an RNA interference component, strongly associates with active enhancers as well as RNA produced at those sites (enhancer RNA, eRNA) [71]. Taken together, these data suggest that enhancer-associated AGO1 contributes to chromatin architecture and gene expression in human cells [71]. Moreover, these studies also revealed that AGO1, in association with NEAT1 lncRNA, contributes to nuclear and 3D chromatin architecture in human cells [71]. Additionally, it has been shown that lncRNA associated with RNA-binding proteins (RBPs) in the nucleus is involved in transcriptional regulation via modulation of 3D chromatin architecture [72,73]. For more data related to long noncoding RNA and 3D chromatin structure see Begolli et al. [74].

### 3.4. Chromosome Fragile Sites in Diseases

CFSs are often involved in chromosomal abnormalities such as deletions, duplications, translocations, and loss of heterozygosity in a number of tumour cells [75,76]. As mentioned earlier, CFSs, in general, are also frequently occupied by miRNAs genes (for more detail see [34]). This was first demonstrated by Calin et al. [34], who showed that over half of the 186 miRNAs studies map to the chromosome regions containing fragile sites. In addition, to confirming Calin et al.’s findings by studying over 700 miRNAs, Lagana et al. [58] demonstrated that the fragile sites are also dense in proteins coding genes. Recently, Kumar et al. [59] have shown that 35.04% of human mature miRNA genes lie within the fragile sites. For instance, fragile sites such as FRA4D (aphidicolin type, common) contain miR-218-1 and FRA5G (folic acid type, rare) contains miR-218-2 [58]. miRNA have also been found to map to the integration sites of human papillomavirus (HPV) [34,77,78]. Additionally, Wang et al. indicated that retrovirus infection induces the expression of the oncogenic miR-17-92 miRNA cluster.

Fragile sites are often associated with multiple neurological diseases and cancers. The most common example of the disease associated with fragile sites is **F**ragile **X s**yndrome (FXS). FXS is linked to the expansion of the CGG trinucleotide repeats, r(CGG), which is associated with transcriptional silencing of either *FMR1* or *FMR2* (Fragile X mental retardation genes 1 and 2) on chromosome X [79,80]. Neuronal stem cells are indeed the hotspots for defective DSB repair, especially in the longer genes [81] leading to many neurodegenerative and neurodevelopmental diseases. The first studied link between neurodegeneration and CFS genes was reported in Alzheimer’s disease (AD) where Sze et al. showed that when downregulated, *WWOX* induces Tau phosphorylation, thus implicating its association to AD [82].

Most studies of miRNAs in cancer have been focused on FRA3B and FRA16D; the two best characterized common fragile sites, which lie within the large tumour suppressor genes. The fragile histidine triad (*FHIT*) gene was isolated from the region encompassing the most active fragile *FRA3B* locus [83]. The tumour-suppressor gene *WWOX*, located within the fragile site FRA16D in chromosome 16q23.3-24.1, is correlated to multiple cancers, especially breast, prostate, and ovary [84,85]. Interestingly, previously mentioned FMR1 also correlates with breast cancer (overexpression of the protein enhances, whereas its downregulation inhibits breast cancer metastasis) [86]. Nevertheless, FMR1 is not only linked to breast cancer since its discovery but also in conjugation with other types of cancer [87].

## 4. miRNAs and Diseases

It is well known that miRNA expression is highly tissue-specific. Some of the miRNAs are even exclusively expressed in a certain cell or tissue types. Therefore, it is not surprising that specific miRNA expression profiles can be identified in different diseases. The deregulation of miRNAs has also been associated with a number of diseases such as hepatitis C virus (HCV) [88,89], immune-related diseases like multiple sclerosis (MS) [90,91,92] and systemic lupus (SL) [93,94], different cancers, and several neurodegenerative disorders. However, miRNA dysfunction has been widely reported in different types of cancers, followed by several neurodevelopmental and neurodegenerative diseases (NDs). Consequently, studies related to miRNAs and their association with cancer and neurodegenerative diseases are discussed in more detail in the following sections.

### 4.1. miRNAs Associated to Cancer

As mentioned, more than 50% of miRNA genes are located at the fragile sites where chromosomal rearrangements associated with cancer occur [95]. Moreover, it has been shown that almost half of the miRNAs are located near or within genes translocated in cancer [58]. Recent studies report that in most cancers, miRNAs are apparently deregulated and under certain circumstances can function as oncogenes (oncomirs) or tumour suppressors [96,97,98]. Aberrant expression of miRNA is directed by different mechanisms. These mechanisms include the miRNA biogenesis pathway, epigenetic silencing as well as genetic alterations, and single nucleotide polymorphism (SNP) [99,100,101,102,103,104,105,106].

The first study that directly suggested miRNA’s dysregulation as an important feature of tumourigenesis came from Calin et al. [107]. They were looking for a gene/genes that could be associated with B cell chronic lymphocytic leukaemia (CLL); however, they failed to identify any protein-coding genes; instead, they found a cluster of two miRNAs, miR-15a and miR-16-1, located at the frequently deleted region in CLL (13q14.3). The expression of these miRNAs was diminished or completely deleted in ≈ 68% of CLL examined cases. Furthermore, researchers identified a germline C-to-T mutation located only 7 base pairs (bp) downstream of the miR-16-1 precursor in two out of 75 CLL patients (mutation not found in 160 control individuals), which correlated with the diminished expression of this miRNA [108].

One of the factors that cause miRNA deregulation is through RNA editing. RNA editing is done by two classes of enzymes. Adenosine deaminase acting on RNAs (ADARs) are responsible for the deamination of adenosine (A) to inosine (I). On the other hand, activation-induced deamination (AID), also known as Apolipoprotein B mRNA editing enzyme, catalytic polypeptide-like (APOBEC; [109]) deaminates cytidine (C) to uridine (U). These RNA-editing enzymes have a significant role in immunity as well as neural plasticity. Moreover, RNA-editing enzymes also act upon miRNA-editing events reported on miR-140, miR-301a, and miR-455 and frequently occur in the seed sequences and in consequence, impact miRNA regulatory functions [110,111]. miRNA editing of miR-376a-1 has been linked to the formation of human gliomas [112]. Similarly, studies done on the samples from patients with bladder, kidney, and testicular cancer also suggest a crucial role in the downregulation of miRNA editing [113,114]. Further studies are necessary to elucidate the importance of miRNA editing in the context of human diseases.

**Figure 1:**
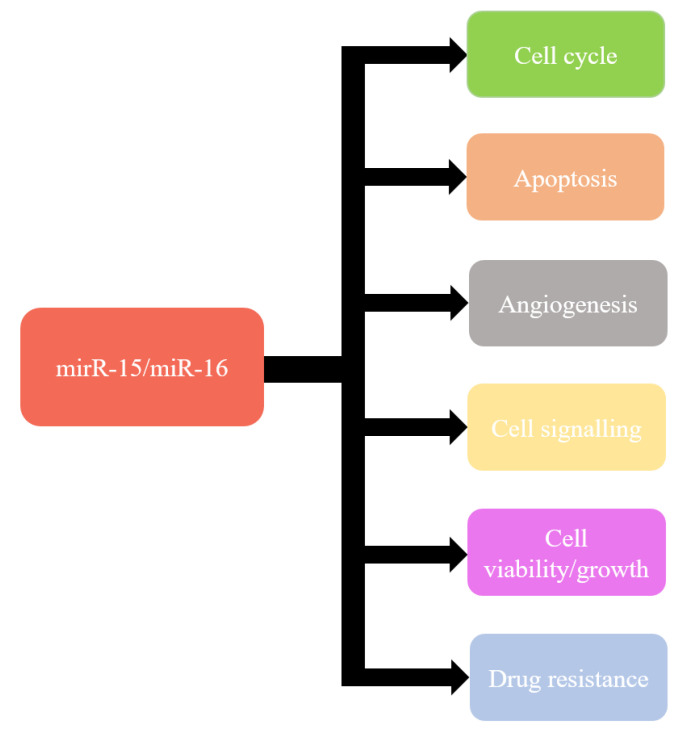
Functions of miR-15/miR-16.

Recent knowledge indicates that miR-15a and miR-16-1 can modulate cell cycle, inhibit cell proliferation, suppress tumourigenicity, and induce apoptosis both in vitro and in vivo [115] (Figure 1). These effects are obtained by targeting the key genes such as *BCL2*, *MCL1*, *CCND1*, *WNT3A*, and genes involved in G1-S transition [97,116,117,118,119,120]. Moreover, miR-15a and miR-16 have also been shown frequently downregulated and/or deleted in other forms of cancer, such as lung cancer, prostate cancer, stomach cancer, pituitary adenoma, multiple myeloma, osteosarcoma, liver cancer, breast cancer, and ovarian cancer [120,121,122,123,124,125].

Through the years, miRNA’s involvement and role were indicated in many types of cancers [126,127,128,129,130,131,132,133,134,135]. The best-characterized tumours and their association with miRNA are listed in Table 1. Since miRNA possesses good stability, high sensitivity, and specificity, it becomes an interesting factor that could be exploited as potential biomarkers. Moreover, inhibition of oncogenic miRNAs or substitution of tumour-suppressive miRNAs serves a potential way for the development of novel treatment strategies.

### 4.2. miRNAs Associated to Neurodegeneration

Similar to their roles in cancer, miRNA editing also leads to neurological disorders [247]. A to I RNA editing can potentially impact miRNA specificity and, consequently, their biological functions in a neuronal cell. For example, Eichler et al. [248] reported that APOBEC-mediated RNA editing is essential in the progression of temporal lobe epilepsy. Similarly, the role of C to U mutations has also been implicated in schizophrenia patients [249]. Deregulation of miR-175 associated with X-linked mental retardation, which also coincides with the early onset of PD [250]. RNA editing and their association with ncRNAs and neurodegeneration are discussed in great detail in the review by Singh [251] and more recently by Lerner et al. [252].

miRNA dysregulation has been reported in a number of neurodegenerative diseases (ND) such as AD, multiple sclerosis (MS), Parkinson’s disease (PD), amyotrophic lateral sclerosis (ALS), and Huntington’s disease (HD) (Table 2). Several other neurological disorders, including schizophrenia, autism, dementia, and epilepsy, have also been associated with miRNA dysfunction.

The hallmarks of the neurodegenerative diseases are neuronal degradation and neuronal death. miRNAs play significant functional roles in several pathways that are critical to neuronal differentiation and survival, making miRNA signatures apparent in several NDs. There have been approximately 600 differentially expressed miRNAs reported in 72 different studies (see Brennan et al. for details) on ND patients with 346 miRNAs identified as unique. The study done by Brenan et al. showed that although many miRNAs were present in at least two ND patient samples, each ND has at least one unique miRNA deregulation [279]. Interestingly, the miRNA hsa-miR-30b-5p overlaps with all four widely studied NDs, i.e., AD, PD, ALS, and MS.

Both overexpression and the downregulation of miRNAs have been implicated with NDs. For example, miR-9 that targets several proteins associated with AD pathogenesis (e.g., BACE1, PSEN1, SIRT1, and CAMKK2) is downregulated in human AD brain samples, as well as in mouse and neuronal cell culture models. On the other hand, the upregulation of miRNAs such as brain-miR-112 and brain-miR-161 has also been reported in the brain samples [271]. Additionally, hsa-miR-30b-5p demonstrated an example where it has been reported to be downregulated in ALS, AD, and PD but upregulated in the case of MS. These reports suggest a profound functional relevance of miRNAs in ND that could only be revealed with extensive studies in the future. Nevertheless, miRNAs prove to have great potential to be targeted for developing biomarkers and therapeutics.

## 5. Therapeutic Potentials of miRNAs

Targeting miRNAs have been gaining attention as a potential tool for the treatment of a number of diseases including cancer and neurodegenerative diseases [280]. miRNA mimics and anti-miRNAs are two popular strategies that are being explored extensively. miRNA mimics are miRNA precursor-like small RNAs currently being developed to regulate the expression of target proteins. In contrast to miRNA mimics, anti-miRNAs are molecules that can interfere and create a loss-of-function for miRNAs of interest [281]. However, like other strategies, miRNAs therapeutic potentials have their share of challenges. More research is required to improve target specificity, efficacy, drug delivery, optimizing off-target effects, etc. One possible and widely researched area of studies in miRNAs’ association with diseases is their potential usage as biomarkers to improve disease diagnosis or prognosis. Using genomic tools for identifying novel miRNAs would more likely give researchers an edge over other currently used methods. Furthermore, FDA-approved clinical tests using real-time quantitative PCRs (qPCR) could be used to amplify low abundant miRNAs for detection. This could prove advantageous, especially because such techniques are not available to measure low abundant proteins or other molecules currently. However, to ensure reliable miRNA measurement, selection of appropriate normalization techniques is equally important [282,283,284].

## 6. Conclusions

**Figure 2:**
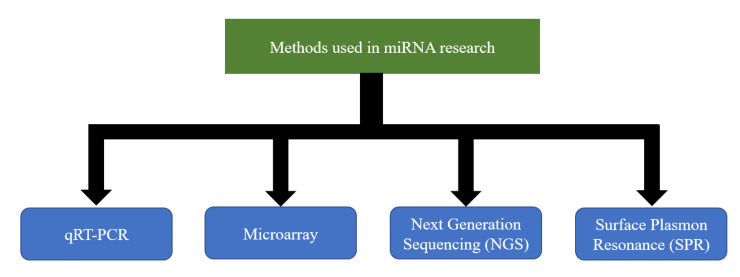
Methods currently used in microRNAs (miRNA) research.

Extensive studies done in the past decades have helped to elucidate the importance of miRNA regulation in the context of a number of diseases and the potential to exploit its use in therapeutics, especially for the so-called incurable diseases. Despite the early success of SPC3649 [285] and the fact that multiple miRNAs have been proposed as potential biomarkers, their use in clinical practice has not been sufficiently materialized. One of the main reasons is the technical challenge of accurately measuring miRNA expression. So far, there does not exist an easy, fast, and inexpensive method that could overcome it. Nevertheless, a number of techniques are currently being used to allow the assessment of the expression levels of the number of miRNAs in a variety of cell types [286,287,288,289,290,291,292,293] (Figure 2). Each technique has its strengths and weaknesses [294]. A strong collaboration between clinicians and researchers with expertise in different techniques would undoubtedly bring different perspectives on the same table that could give the boost required for the steady development of clinical applications.

## Figures and Tables

**Table 1:** Cancers and their association with microRNAs (miRNA).

Type of cancer	Micro RNA	Reference
Chronic lymphocytic leukaemia	miR-15a, miR-16-1, miR-21, miR-27b, miR-29a, miR-34, miR-91, miR-95, miR-144, miR-155, miR-181,	[107,136,137,138,139,140,141,142,143,144]
Breast cancer	miR-10b, miR-16, miR-21, miR-27b, miR-29a, miR-34, miR-106a, miR-125b, miR-126, miR-145, miR-155, miR-199a, miR-210, miR-335, miR-589, let-7c	[124,133,142,145,146,147,148,149,150,151,152,153,154]
Gastric cancer	miR-145-5p, miR-29c, miR-200, miR-18a, miR-96, miR-107, miR-148a, miR-181a, miR-300, miR-370, miR-421, miR-520-3p, miR-600, hsa-miR-29b-1-5p, has-miR-27b-5p	[147,155,156,157,158,159,160,161,162,163,164,165,166]
Prostate cancer	miR-15a miR-16, miR-29b, miR-30c, miR-34 miR-16-1, miR-141, miR-221, miR-222, miR-335, miR-375	[120,167,168,169,170,171,172,173,174,175,176,177,178,179,180,181,182,183]
Liver cancer	miR-29, miR-34, miR-101, miR-122, miR-145, miR-195, miR-214, miR-370, miR-375,	[184,185,186,187,188,189,190,191,192,193,194,195,196,197,198,199,200,201,202,203,204,205,206,207,208,209,210,211]
Lung cancer	miR-15a, miR-16, miR-21, miR-27a, miR-29, miR-30b, miR-30c, miR-34, miR-101, miR-125b, miR-126, miR-130b, miR-132, miR-134, miR-135b, miR-153, miR-155, miR-182, miR-192-5p, miR-195-5p, miR-196, miR-200b, miR-205, miR-210, miR-212, miR-218-5p, miR-449a, miR-494, miR-520a-3p, miR-641, miR-660, miR-760, miR-1258, let-7, let-7a,	[121,212,213,214,215,216,217,218,219,220,221,222,223,224,225,226,227,228,229,230,231,232,233,234,235,236,237,238,239,240,241,242,243,244,245,246]

**Table 2:** Neurodegenerative diseases and their association with miRNA.

Disease	Micro RNA	Reference
ALS	hsalet-7a-5p, hsa-miR-1, miR-206, miR-143-3p, miR-374b-5p, hsa-miR-760, hsa- miR-744-5p	[253,254,255,256]
PD	miR-7, miR-16-1, miR-34 b, miR-34c, miR-153, miR-138-2-3p, miR-205, miR-224, miR-320a, miR-373, miR-379, miR-4639-5p, miR-494	[257,258,259,260,261,262]
AD	miR-9, miR-29, miR-34, miR-101, miR-107, miR-124, miR-153, miR-181, miR-195	[263,264,265,266,267,268,269,270,271]
MS	hsalet-7d-3p, hsa-miR-122-5p, hsa-miR-125a, hsa-miR-125b	[272,273,274]
HD	miR-9, miR-196a, miR-132, miR-10b-5p	[275,276,277,278]
